# Notes and Comments

**Published:** 1922-08

**Authors:** 


THE
### HOSPITAL and HEALTH
REVIEW

CORRESPONDENCE :—

Maternity Homes : The Henry Saxon Snell Prize—A Central Fund for Provincial Hospitals—Home Nursing for Insured Persons . . . . . 338

### HEALTH IN THE NEAR EAST.

EARLY this year a Commission appointed by the Health Organisation of the League of Nations visited the Suez Canal and those ports whose sanitary services fall under the Sanitary Maritime and Quarantine Board of Egypt, then the Red Sea, returning via Jerusalem and Beyrout across the Ægean to Constantinople. Its object was to inquire into the international arrangements regarding the prevention of epidemic disease in the Near East. The representative of Great Britain was Sir George Buchanan, Senior Medical Officer of the Ministry of Health. The report of this Commission has recently been issued, in French and in English, by the League of Nations.

Much of the report is technical, showing the modifications proposed in Parts I. and III. of the International Sanitary Convention of 1912. This document seriously needs revision. Ten years ago commercial considerations, as much as those of public health, influenced its clauses. The Commission, composed of practical sanitarians, has made important recommendations which the Governments responsible ought to enforce at the earliest possible opportunity.

The report falls naturally into four divisions. There is, first, the question of the sanitary barrier necessary around the Suez Canal, which is the great highway of shipping between East and West. No alteration is contemplated in the administration that exists, with the exception that, owing to the opening up of railway communications between Egypt and Palestine and Asia Minor, special measures are needed against infectious diseases being imported by railway. The second part of the report concerns the annual gathering of pilgrims at Mecca. The difficulty here is for non-Moslem countries to enforce sanitary measures within the Hedjaz. Practical proposals are made, however, to keep pilgrims, both before and after their visit, under strict supervision. The third part deals with epidemic diseases in Syria and Palestine. There is at present considerable overlapping, as shown by the fact that Sir George Buchanan's ship, in travelling from Beyrout to Constantinople, within nine days received nine visits from the medical representatives of the French, Turkish, British, Italian and Greek Governments, as well as at Chanak from the Inter-Allied representatives. These inspections were made under different regulations, and the report urges that this unnecessary expenditure of time and money might be avoided by local agreements among the different sanitary authorities.

The fourth part of the report deals with the position of Constantinople, where at the present time there is a lack of sanitary accommodation for cases of infectious disease. This is a danger-spot of the Western world, owing to the prevalence in the Black Sea ports of typhus, relapsing fever, smallpox and cholera, and the continued flow of refugees from South Russia to Constantinople. The report pays a tribute to the excellent sanitary work accomplished during the Inter-Allied occupation. This is, however, only temporary, and it is urged that a Port Sanitary Authority should be appointed.

The work that is being done in various parts of the world, largely inspired by one or two men of vision like Sir George Newman and Sir George Buchanan in this country, Dr. T. H. Madsen, of Denmark, and Dr. Violle, of France, would make a romantic story if all the details could be given. More than romantic, it is an essential work, safeguarding Western Europe from diseases that in the Middle Ages were the scourge of mankind. The British Government has recognised the value of the work by making a grant-in-aid of £100,000.

### THE MULTIPLICATION OF MENTAL DEFECTIVES.

MENTAL deficiency is hereditary, and those who suffer from it are prolific—more prolific than the general population, as the restraints which check fecundity do not come into play. Their multiplication is not only a burden, but tends to deteriorate the race. In a series of articles this evil has been discussed in the "Morning Post," and the suggestion of Sir Archibald Reid, that all mental defectives should be sterilised and thus rendered incapable of propagation, is recommended. A correspondence, in which many well-known persons joined, shows that public opinion in this country is against such a remedy. In the United States legislation has gone to the extent of permitting compulsory sterilisation, but it is not employed. The patient is consulted, or, if his mental powers do not allow this, the question is submitted to his relatives. Dr. A. F. Tredgold, a well-known expert on mental deficiency, points out that segregation is just as efficient as sterilisation and has other advantages. All mental defectives are incapable of maintaining existence and at the same time conforming to the laws of the community without some amount of control, either for their own protection or the protection of others. Many are centres for the spread of venereal disease, and many commit crimes. Hence they should be detained in institutions even if they were sterilised. There are thousands of defectives in this country who, either for their own sakes or for that of the public, should be in institutions, and the failure to provide accommodation is an extremely short-sighted policy. Dr. Tredgold does not consider it possible to apply sterilisation to all grades of mental defect without the risk of including non-defectives. In the case of idiots and imbeciles diagnosis is easy and such risk does not exist. But these classes comprise less than one-fourth of the total number of defectives, and many of them are naturally sterile, and, therefore, do not need the operation. The great danger of propagation is in the case of the feeble-minded, to which grade fully three-quarters of all defectives belong and which is highly prolific. Diagnosis in these cases may be very difficult. During the unstable period of adolescence there are many varieties of mental abnormality, readily confused with defect, which are often temporary and amenable to treatment. There is, moreover, a very large class, known as "border-landers," who occupy a position between defectives and the normal. While considering sterilisation justifiable and advisable in particular cases of defect, Dr. Tredgold points out that it does not remove the necessity for segregation and institutional care in the majority of cases.

### THE PREVENTION OF VENEREAL DISEASE.

AT the Plymouth Congress of the Royal Institution of Public Health, the Society for the Prevention of Venereal Disease promulgated a detailed policy which, if carried out, should do much to stamp out the scourge of venereal disease. The Society takes its stand on three main principles. (1) The duty of chastity for every good citizen. (2) That it is the office of medical science to prevent the consequences of sexual immorality. There is no doubt that venereal disease is a cause of widely spread suffering. The Royal Commission on Venereal Disease made the following statement: "The evidence we have received leads us to the conclusion that the number of persons who have been infected with syphilis, acquired or congenital, cannot fall below 10 per cent. of the whole population in the large cities, and the percentage affected with gonorrhœa must greatly exceed this proportion." It is impossible to overestimate the evil. For the tragic fact is that the consequences are not limited to the guilty, but pass to innocent women and children. (3) That venereal disease can be prevented by immediate self-disinfection, provided it is intelligently applied. Immediate self-disinfection, like many other preventive measures, may be occasionally inoperative through misuse or delay.

The Society looks with favour upon all curative measures, and heartily approves the institution of venereal clinics. It holds that instruction in methods of self-disinfection should not be refused to men who have reached adolescence, to adult women if they desire it, or even to younger persons at the discretion of their parents or those responsible for their welfare. It advocates the propagation of such knowledge as may tend to diminish venereal disease and its grave consequences by lectures and leaflets, assuming that in general the lectures are attended and the leaflets received by such persons only as have reached the age of adolescence. It wishes also to keep an open mind for all such social and medical developments as may tend to the extirpation of the scourge which has so long afflicted humanity.

The policy of the Society may be summarised as follows :—

1. To instruct the public as to (*a*) the great importance of self-disinfection, at the time of exposure to risk, as a preventive of venereal disease, and (*b*) the methods of application of such disinfectant.

2. That such instruction should be given only to men above the age of 18, to adult women on demand, and to younger persons in special cases at the discretion of those responsible for their welfare.

3. To ensure the sale of the disinfectants required, together with full instructions, to such persons, but to such persons only.

4. That instruction regarding the nature and application of such disinfectants should be conveyed to such persons : (i.) By leaflets supplied (*a*) with the disinfectants at the time of sale, and (*b*) by attendants at public conveniences on demand; (ii.) by lectures; (iii.) by medical officers of venereal clinics; (iv.) by officers or officials in charge of naval, military, and industrial units.

5. To advocate such further steps for the prevention of venereal disease as may from time to time be deemed advisable by the executive committee.

### THE NATIONAL COUNCIL FOR COMBATING VENEREAL DISEASES.

AT the seventh annual general meeting of the National Council for Combating Venereal Diseases, at the Barnes Hall, Wimpole Street, Lord Gorell, the retiring president, reviewed the position and said that the Council had no cause for dissatisfaction with it. A year ago it had been necessary to devote a presidential address to exposing a campaign of misrepresentation against the National Council, but the Council now stood in an unchallenged position. The setting up of Lord Trevethin's Committee of Inquiry carried out the policy that the Council had advocated for some time. Lord Gorell was grateful to Lord Dawson, who had done so much towards securing the appointment of this Committee, for the courteous way in which he had received suggestions. He hoped that the Committee would be successful in finding a *via media* in which all who were genuinely solicitous for the national welfare could meet and agree. He deprecated the continuance of a technical controversy, which was not only misleading to " the man in the street," but caused the dissipation of much energy and resource. The Ministry of Health believed that the time had not arrived for any general system of compulsory notification of venereal diseases in this country, and there was much to be said for this point of view, but indications were not wanting that before long the country would demand the protection of its innocent citizens and children, by means of legislation penalising those who discontinued treatment while still infective. Lord Gorell referred to the systems now operative and the results obtained in some of the overseas Dominions and foreign countries. At home the work of the medical profession, the hospitals and the clinics was heavily handicapped by the lack of administrative measures in this matter of continuous treatment. The question was not to be confounded with any system of regulated prostitution; it was a public health matter which must be dealt with as such, and in no other way. In countries where this attitude had safeguarded the framing and administration of the law, the results were undoubtedly conducive to the public welfare.

### FIGHTING INSECT-BORNE DISEASES.

SIR ARTHUR SHIPLEY, the well-known entomologist, contributes to " The Times " an interesting account of the Imperial Bureau of Entomology, which was established in 1913 to encourage and co-ordinate entomological work throughout the Empire in relation to human, animal and plant diseases. The committee is widely representative and includes the chief entomologist in each of the Dominions. The extent of the work done is shown by the fact that during the past year some 53,000 insects have been submitted for identification by 82 correspondents scattered throughout the Empire. This collection often contained, of course, more than one specimen of a single species. Identifications of 4,300 different species have been issued in the same period. Much of the material is returned to the collectors, but 13,000 specimens were presented to the British Museum, of which 171 were types or species new to science, and 428 were species not previously represented in the national collection. Some 5,000 named blood-sucking insects were distributed to various institutions throughout the Empire for teaching purposes.

The Bureau issues two valuable periodicals. One, a quarterly, is a record of original research. The other, the " Review of Applied Entomology," is published monthly in two series, one dealing with insects injurious to plants, the other with insects that disseminate human and animal diseases. It summarises all the papers or books concerning insects of economic importance published in any part of the world in any language. The Bureau has many other activities. It has collected a library of the utmost value, and has prepared an analysis of the laws of the various countries with respect to the importation of plants which may bring with them new and dangerous pests. It has sent students to study the methods of dealing with insect pests on a large scale in the United States. It has also sent out to Tropical Africa investigators who have done good work in connection with disease-carrying insects under trying circumstances. The committee have under consideration a new activity—the supply of beneficial parasites to keep down insect pests.

### POISONED FURS.

AS early as 1913 Prof. Blaschko, of Berlin, drew attention to the dangers of paraphenylendiamin —a violent skin irritant—used as a dye in the fur trade, but it would seem that his warning has fallen on deaf ears. This substance is used for converting rabbit's fur into " brown beaver," and produces a most successful imitation. But when such fur is wetted, the irritant properties of paraphenylendiamin, or its oxidisation product, chinondichlordiimin, are enhanced, and the results are apt to be most unpleasant. Recently in Copenhagen, Dr. C. Rasch has observed nine cases of severe inflammation of the face and neck which he has traced to this source, and he refers to similar observations by other Danish doctors. His patients complained of great discomfort due to the itching, burning and actual pain provoked by the dermatitis of the face and neck. All Dr. Rasch's patients were women who, a few weeks or months earlier, had invested in brand-new coats trimmed with beautiful brown fur, and some of them had suffered no discomfort therefrom till they had been out of doors for several hours with their fur collars turned up. The reaction provoked varied so greatly in different cases that it is creditable of Dr. Rasch that he traced them all to one and the same cause. In two cases the reaction was a constantly recurrent itching redness on the chin. In other cases an eczematous eruption appeared, and the face of one patient was disfigured by a vesicular erythema and swollen lips. In another case the eyelids were itching and swollen. There could be little doubt as to the cause, for in every case on discarding the furs all the symptoms disappeared in five to fourteen days. The fur in almost every case was a preparation known to the trade as "biberette," and it is fairly safe to venture the guess that, in Copenhagen at any rate, there is a bad slump in "biberette" just now. The question naturally arises: How many cases of poisoning by dyed furs have occurred in the past without the origin of the rash coming to light? If the truth were known, we should probably find that the dermatitis of faked furs is quite common, and that it has masqueraded under various disguises, and has been treated with an equally great variety of liniments, pills and potions.

### TRAINING FOR IMBECILE CHILDREN.

THE Central Association for Mental Welfare, 24 Buckingham Palace Road, S.W.1, has issued an illustrated booklet on the organisation of centres for the occupation and training of mentally defective children. The object of these centres is to provide for those mental defectives in urban areas and small towns for whom no other form of education is available. It is the duty of Local Education Authorities to provide special day schools for feeble-minded children, but those of a lower grade (imbeciles) are not admitted to such schools. The Occupation Centre is mainly intended for them, but may admit children who are only feeble-minded if there is in the area no Special School to which they can be sent. It may even take defectives over 16 who are so childish and undeveloped that they can be safely mixed with the others. By providing the defective child with opportunities for learning muscular control, cleanliness, obedience and simple manual work, such a Centre, even if open only two or three days a week, can so stimulate him that real mental and moral development results.

Most of the Centres already established have been started by the local Voluntary Association for the Care of the Mentally Defective, but there is nothing to prevent any other group of workers from undertaking the work, provided that they obtain the co-operation of the Committee for the Care of the Mentally Defective (a Statutory Committee of the County Council or the County Borough Council, appointed under the Mental Deficiency Act) and of the Local Education Authority. The Central Association is prepared to send, free of cost if necessary, an experienced member of its staff to help the responsible local worker with the preliminary organisation.

The cost of the Centre mainly depends on whether or not a paid teacher is employed, but many Centres are successfully run by voluntary workers. It is pointed out that this is one of the few branches of mental deficiency work which can be suitably undertaken by quite young people, and that a special effort should be made to enlist their sympathy and help. A suitable room can usually be obtained rent free, at the local school clinic, settlement or guild of help, or in a hall or church room which is only used at night. A centre open on two or three half-days a week and attended by seven or eight children has been carried on for as little as £12 a year.

### PUBLIC HEALTH AND THE HOUSE-FLY.

FOR many years the York Health Department has carried on a vigorous annual campaign against the house-fly. Leaflets on this pest are distributed by sanitary inspectors and nurses in the houses which they visit, and are circulated in shops and restaurants. In the schools pictorial posters showing the evil habits of the fly are expounded to the children, who take home leaflets, and in other ways have proved themselves to be excellent missionaries in this cause. Picture palaces help by exhibiting special films. The Health Department pays special attention to manure heaps, to the diminishing number of privy-middens and brick ashpits, and to uncovered refuse bins. It appeals to shopkeepers to cover with muslin food, milk and other things exposed for sale that attract flies, and to owners of stables, etc., to see to the removal of manure and other refuse heaps, at least once every week. It advises the household burning of all waste matter and the protection of milk and other food. Suitable disinfectants for use in ashpits, etc., can be obtained free on application at the Health Department.

That these measures have had good results is proved, not only by the absence in recent years of the plagues of flies once experienced in hot summers, but by the decrease of typhoid fever and summer diarrhœa. During the last three years an average of only six genuine cases of typhoid fever were notified in the city, as compared with an average for the five years 1909-1913 of 32, for 1904-1908 of 61, and for the year 1900 a total of 244. In the hot year of 1921 there were 33 deaths from summer diarrhœa amongst children under two years of age, as compared with 10 in 1920, an average of 73 within the five years 1904-1908, and 148 in the year 1900.

### ICE CREAM.

SHOULD August provide seasonable holiday weather, without doubt ice cream will be consumed in large quantities by many people who do not realise that the ice cream question is becoming a serious one from the health point of view. Of recent years in this country the consumption has been steadily increasing, although it has by no means reached the extent of the demand in America. Here we are content with the ordinary dab of so-called ice cream, made possibly of cornflour and milk preserved for too long a period, served up with some chemical flavouring. In America the provision of ice cream has become a business above the standard of the hokey-pokey merchant. There the nutritive value of this delectable food, provided that it is made from fresh cream and milk, is widely advertised, and it is served up in a variety of attractive forms, varying from " Eskimo pie " to " cornets."

One or two far-seeing firms in this country are at last beginning to try to develop an ice cream business. A visit to one of these factories showed how it is possible to manufacture ice cream without its being touched by human hand, from the time when the buckets of pure cream are poured into the machines until the solid blocks are taken out of the refrigerating chamber. In such a case the flavourings are not mere chemical mixtures, but the natural fruit, sent over from France and other countries that make a speciality of bottling strawberries and raspberries expressly for the ice cream trade. Not a quarter of a mile from this factory was situated an insanitary room, overlooked by a stable, where much dirty and unwholesome ice cream was manufactured until the place was closed by an alert sanitary inspector.

At present no standard appears to have been set up by the Ministry of Health to defin: this delicacy. Apparently no one is allowed to manufacture ice cream in a bedroom or to keep it under a bed, but broadly speaking, except for such rules, there is no protection for the public. Experience has shown that one satisfactory way in which the public can assist in remedying or raising the standard is by asking to be allowed to see the place of manufacture. The more reputable firms are always ready to welcome visitors to see the process. Only those who know that the place of manufacture is too insanitary to bear inspection object to visits from their customers.

### HARMFUL PRESERVATIVES IN FOOD.

A NUMBER of medical, scientific, philanthropic and labour societies, including the British Medical Association, the Society of Medical Officers of Health, the Royal Sanitary Institute, the National League for Health, Maternity and Child Welfare, the National Association for the Prevention of Tuberculosis, the Bread and Food Reform League, the General Council Trades Union Congress, and the Medical Committee of the Industrial Welfare Society, have addressed an appeal to Members of Parliament urging the need for protection of food from adulteration and deterioration, with special reference to the excessive use of preservatives. Over 90 M.P.s, representing the medical profession and all parties in the House of Commons, have signed a manifesto stating that they will do all that they can to obtain for Great Britain the protection against food adulteration that has been achieved by Australia, Canada and New Zealand, and to support legislation for securing pure and nutritious bread. The Dominions provide that distinct notification shall be given to the purchaser of the nature and amount of any chemical preservative contained in any food sold for human consumption. The public are thus able to decide for themselves if they will use food containing possibly harmful drugs. Food inspections show that the list of articles known to contain boric acid is extending, and that such foods are frequently consumed by infants and invalids, on whom they may have a specially injurious effect. Parliamentary assurances have been received that the attention of the Government will be directed to practical suggestions for dealing with this danger. Further particulars can be obtained from Miss May Yates, hon. secretary, Educational Health and Food Campaign, 37 Essex Street, London, W.C.2.

### "HANDY WOMEN."

THE recent deaths of mothers in childbirth apparently due to the inefficiency or carelessness of the " handy women " attending them have again raised the question of the necessity for the more effective administration of the Midwives' Act of 1902. Under this Act an uncertified woman is prohibited from attendance for the sake of gain upon mothers at the time of their confinement, " except under the direction of a qualified medical practitioner."

Several difficulties occur in carrying out this requirement. There are cases where neighbours come in and help in case of emergency, but if they do not do so for the sake of gain they are not affected. In other cases the patient herself is prejudiced in favour of some local " handy woman " with whom she is on friendly terms, in spite of her possibly unclean methods. There are cases where the practitioners themselves are at fault. They only pay perfunctory visits to the patients and allow the " handy women " in many instances to deliver the child. In fact, the words " under the direction of " are very loosely interpreted in many quarters.

The General Medical Council in 1916 issued a very strongly worded notice to medical men, pointing out that the proper working of the Act was of the utmost importance. But recent examples make it clear that in too many cases this warning has been disregarded and the law defied.

This is certainly a matter that the Central Midwives' Board ought to consider in all its bearings. There is a growing feeling that some system of inspection ought to be introduced. The certified midwife works under proper supervision, but the death-rate of mothers during the last three years has proportionately increased. It is of little avail to have regular inspection of the work of midwives, if in many cases their skill is not being used and untrained " handy women," undirected and unsupervised, attend to mothers. It is understood that this whole question is causing anxiety in responsible quarters, and it is hoped that some action will be taken as soon as possible.

### PANEL DOCTORS' ABSENCE.

DR. WALDO, the Coroner for the City of London and Borough of Southwark, at a recent inquest stated that in the present panel system there were many defects opposed to the vital interests of the public. One was the absence of many panel doctors from their houses and surgeries at night, with the consequent difficulty of obtaining medical advice in sudden and dangerous illnesses. He was of opinion that panel doctors should either leave an assistant on the premises at night, or make an arrangement for a colleague to attend patients in urgent cases. So far as he could judge, panel doctors were doing extremely well for themselves. Many of them, unlike coroners, could now afford the luxury of a motor car and two houses. This question of the insurance practitioner living in certain cases some miles from his surgery is receiving the attention, so it is understood, of the officials of the National Insurance Department. By his contract the practitioner is bound to be within reasonable call of the patients on his panel, and action can be taken against those who live too far away. From recent evidence it would certainly appear that much more drastic action ought to be taken. Considering the comparatively large income now being made by insurance practitioners, it is a matter of national importance that they should carry out their work efficiently. The majority endeavour to fulfil their responsibilities loyally, but there is a minority whose conduct arouses numerous complaints. The Minister of Health has certain powers at his disposal, and certainly should use them whenever adequate reason is proved in the case of a panel doctor who does not give proper attention to his patients.

---

### TYPHOID EPIDEMIC DUE TO A POLLUTED WATER SUPPLY.

AN epidemic of enteric fever, which occurred in 1921 at Bolton-upon-Dearne, in the West Riding of Yorkshire, is the subject of a report* by Dr. W. V. Shaw, a Medical Officer of the Ministry of Health. The district affected consists of mining villages, and there were 351 cases of enteric fever, with 40 deaths. After careful inquiry it was discovered that the only factor common to the persons attacked was the consumption of water pumped from the Dearne Valley Colliery. Although no definite cause of its pollution was discovered, it appeared probable that during the dry weather of last year the surface of the ground became baked and cracked, and that a shower of rain carried polluting material from the surface to lower levels. The report contains most useful information as to the action taken to limit the spread of the disease in both outbreaks, and suggests methods of treating water liable to be contaminated in such a manner that no undue risks will attend its consumption.

Sir George Newman, in an introduction, insists that this outbreak of widespread and fatal illness is a warning against being lulled into a false sense of security. " Quite apart from the occurrence of typhoid and other endemic diseases transmissible by drinking-water, cholera has at the present time assumed epidemic proportions in Russia and Poland, and the possible introduction of the infection of this disease into England is a fact that has to be realised." This Yorkshire community suffered severely in sickness and in death from a disease which was preventable. It is the duty of other local authorities responsible for water supplies to take effective action to prevent similar outbreaks from occurring this year.

* " Reports on Public Health and Medical Subjects, No. 12. Dr. W. V. Shaw's Report to the Ministry of Health on an Epidemic of Enteric Fever at Bolton-upon-Dearne." (H.M. Stationery Office ; 2s. net.)

### THE FREQUENCY OF CANCER IN OLD AGE.

THERE is a popular belief that the incidence of cancer declines in extreme old age ; the very old are supposed to be too tough even for malignant disease. They gradually fade away, but do not die of cancer. There is, of course, no such immunity, and one reason why cancer has come to be considered rare in old age is that the number of persons over three score years and ten is comparatively small. Another reason, little appreciated, is that cancer is often overlooked in the aged, and found by accident post mortem. Statistics illustrating this point have lately been published by the pathologist attached to the large communal hospital of Christiania, where, in the 10-year period 1909-1918, 710 cases of cancer were found post mortem. In as many as 113, the cancer had not been diagnosed during life.

It is not flattering to the efficiency of modern diagnostic methods available in a large, up-to-date hospital that 15.9 per cent. of all cases of cancer should escape detection before death. Among the persons found post mortem to be suffering from cancer there were 56 over the age of 80, and in as many as 46.4 per cent. the cancer was unrecognised before death. If in hospital practice almost every other case of malignant disease in the aged escapes detection during life, what is the proportion of undetected cases in general practice when the patient is visited only once or twice by a practitioner ? It must surely be great. It is instructive that among the cases of cancer occurring in persons under the age of 60, the disease was overlooked only in 7·4 per cent. The difference between this figure and the above-mentioned 46·4 per cent. is remarkable.

---

### SUNLIGHT IN THE PREVENTION OF RICKETS.

THAT sunlight has an invigorating effect on the human body has long been known, and in recent years its value in the treatment of disease (heliotherapy), particularly in tuberculosis, has been recognised. In the life of the plant the sun's rays are of much greater importance than in the life of the animal, for they supply the energy necessary for the building up of the tissues from the simpler constituents of the soil and atmosphere. In the plant are formed vitamines, recently discovered bodies present only in traces in our food and yet essential to health. It is generally believed that vitamines cannot be produced in the animal.. One of the causes of rickets is deficiency of " vitamine A," and cod-liver oil is curative because it contains a large supply of this body. Now American observers have shown by experiments on animals, fed on diet which produces rickets, that the disease can be prevented by exposure to sunlight. Thus we have the curious fact that administration of the vitamine and exposure to the sun have the same effect. To explain this, it has been suggested that exposure to the sun enables the animal, as it does the plant, to produce the vitamine—a most interesting assimilation of animal to plant physiology. The hygienic importance of the discovery is obvious.

### THE AXE AT NEWCASTLE.

AN interim report has been issued by the committee appointed last September to inquire into the possibility of economy in the finances of the Newcastle Corporation. Since 1911 the Corporation rates had risen from 6s. to 14s. in the pound; and since the Health Department is one of the principal spending departments it is the first to be dealt with in the interim report. Since 1911 its cost has risen from £1,179 to £3,671, an increase of 211 per cent., and the cost of the Infectious Diseases Hospital from £210 to £1,649. In this period the number of cases has risen from 31 to 47 only. The committee therefore regard the increase as excessive, and recommend that the hospital be closed and the patients distributed among neighbouring authorities, who have been approached satisfactorily. The hospital buildings, the committee suggests, could be used, without structural alteration, for a handicraft and school centre for feeble-minded and delicate children for whom the Council will shortly have to provide, and for whom a grant is payable by the Board of Education. Among other recommendations and suggestions the committee declares that the expense of laying the lawn at the hospital was so heavy that the matter should have been submitted to the Council. The committee also views with alarm the growing cost of the maternity and child welfare department, and recommended that the lease of the Health Offices be not renewed, that the temporary clerk in these offices be dispensed with, and that the Medical Officer of Health be asked to report upon the administration of the welfare scheme.

In a word, the committee doubts whether the amount of new work undertaken by the Corporation justifies the recent rapid increase in its cost, and its report bears every sign of careful consideration. It does not appear that the recommendations, if adopted, need contract the work to be done. The economies proposed are mainly administrative, and since the necessity for an isolation hospital depends upon local conditions, the economy committee at Newcastle has much less to fear from sound criticism than many others which are penny wise and pound foolish regarding the cost of public health departments.

---

### THE PROBLEM OF THE BOARDED-OUT LONDON CHILD.

TO send weakly London children to the country may seem so desirable that the problems of their presence are not always realised in town. A Sussex Rural District Council provides an example. Of a few cases of scarlet fever in the district, at least one was a child who had been boarded out locally by the London County Council. Discussing the possibility of recovering from the London County Council the cost of this child's maintenance in the local Isolation Hospital, a member complained that the local Council had no control over any cottager willing to take a London child, though the local Council has to be responsible for it, to find school accommodation for the lot, and to pay any hospital charges arising from the disease, which, he said, the children often bring. The Clerk explained that the London County Council had power but could not be compelled to pay for the hospital charges. Presumably, no child incubating infectious disease is wittingly sent from town, and, if due precautions are taken, illness, if it occurs, is acquired locally. It should be possible to make a working agreement whereby in each case local or London responsibility could be determined; since no local Council, however rightly anxious to be protected from invasion by infectious disease, would wish that weakly children should be deprived of country air merely because they may fall ill or require school accommodation. If London will be responsible for infectious diseases among these children, the local authorities might be responsible for schools and all else.

---

### A "RAT BARRACK."

DR. J. A. HARAN, C.M.G., in the "British Medical Journal," has called attention to an ingenious method of destroying rats: Whilst looking over Hall's "Ireland" (London: How and Parsons, 1841) he happened upon the following: "Mr. Russell has a 'rat barrack' on his premises. It is about 12 ft. long and 6 ft. broad, and the walls about 4 ft. high, with a coping stone on the top that projects a couple of feet inside the wall—the inside of the wall is full of holes that just admit a rat's body, leaving his tail outside—the whole is covered with old boards; there are two passages for them to come outside into the yard, where they are fed and never disturbed; the consequence is they never go into his store where the bacon is; once every three months he closes the holes that communicate with the yard—he uncovers the walls, and the rats all run into the holes in the walls; their tails are 'hanging out,' when a man goes in, takes them one by one by the tails and throws them into a barrel, when they are all destroyed, to leave room for a fresh supply."

---

### THE DISCOVERY OF THE TYPHUS GERM.

DR. N. KRITCH, the woman director of the Sokolnichersky Hospital Laboratory, is reported to have discovered the germ of typhus. She recently described her work before a meeting of the bacteriological section of the Moscow Medical Society. In collaboration with Dr. V. Barikan, director of the Microbiological Institute, Moscow, she has been working on the etiology of typhus fever since 1916. She has succeeded in isolating a disc-shaped coccus, in appearance much like the pneumococcus. The germ was found in the brain tissue and spleen of 150 cases of typhus one hour after death. The organism was then grown in a sterilised emulsion of pancreatinised spleen. Inoculation of guinea pigs with it invariably produced the symptoms of typhus fever. If the facts are correctly reported, the discovery of an effective serum for typhus should follow before long, when we may hope to control the scourge now almost unchecked in Eastern Europe.

